# Immunoglobulin G4‐related disease: a rare disease with an unusual presentation

**DOI:** 10.1002/ccr3.583

**Published:** 2016-06-01

**Authors:** Muhammad Waqas Khan, Terrance Hadley, Melissa Kesler, Zartash Gul

**Affiliations:** ^1^ Department of Internal medicineDivision of Hematology/Bone Marrow TransplantationUniversity of KentuckyLexingtonKentucky; ^2^ Hematology/Oncology DepartmentNorton HealthcareNorton Cancer InstituteLouisvilleKentucky; ^3^ Department of Hematopathology, Pathology and Laboratory MedicineUniversity of KentuckyLexingtonKentucky

**Keywords:** Fibro‐inflammatory disease, IgG4‐related disease, IgG4‐associated multifocal systemic fibrosis, IgG4‐positive multi‐organ lymphoproliferative syndrome

## Abstract

IgG4‐RD can also present in the skeletal muscle, mimicking several other diseases. It is unusual for this relatively new classification of diseases to present in the muscles and can be mistakenly diagnosed as other autoimmune diseases rendering a delay in the appropriate management and progression of the disease.

## Case Report

A 68‐year‐old lady of an African American descent presented to the office with complains of unintentional weight loss of thirty pounds over the last year. She had a history of follicular lymphoma that had transformed to a diffuse large B‐cell lymphoma (DLBCL) diagnosed more than 20 years ago. It was treated with eight cycles of chemotherapy (cyclophosphamide, daunorubicin, vincristine, and prednisone) followed by radiotherapy. She underwent complete remission with no evidence of recurrence until now.

She was noted to have pancytopenia in September 2014. Owing to a concern for the recurrence of lymphoma, a positron emission computerized tomography (PET–CT) was obtained which revealed a mass in her psoas muscle. Biopsy of the mass showed the presence of a few macrophages and failed to reveal any evidence of malignancies or reoccurrence of lymphoma.

**Figure 1:**
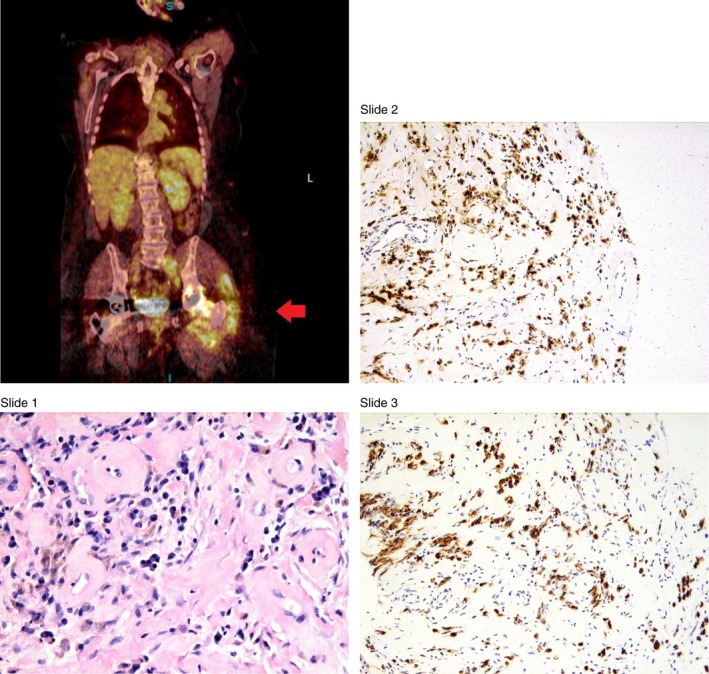
PET–CT image of psoas muscle. Histopathology slide 1: Hematoxylin and eosin‐stained Psoas muscle biopsy 400× magnified. Histopathology slide 2: Immunohistochemistry showing light chain Lambda immunoglobulin 200× magnified. Histopathology slide 3: Immunohistochemistry showing Kappa immunoglobulin 200× magnified.

The patient then started noticing further weight loss and decrease in her appetite since, and presented to the office again after a year. A repeat PET–CT was performed which demonstrated an overall enlargement of the left iliac and psoas muscles (Fig. 1). The FDG‐uptake (fluorodeoxyglucose ‐uptake) along the left rectus femoris muscle had increased to 12 from the previous value of 9.2. The thickness had also increased to 3 cm from the previous 2 cm last year. A new area of uptake was also noted in the left gluteus medius muscle that had an uptake value of 7.9.

Core biopsy of the masses performed thereafter revealed fibrosis with sparse lymphocytic infiltrate and hemosiderin deposition, unremarkable plasma cells, without evidence of a lymphoma (histopathology slides 1–3). A quantitative IgG was performed that showed a value of 2030 mg/dL (reference range: >19 years of age 700–1600 mg/dL). IgG4 subclass was also significantly elevated at 1022 (reference range 7–89). Workup for multiple myeloma was negative with serum protein electrophoresis showing a chronic inflammatory pattern and did not identify a monoclonal protein, but rather a polyclonal increased in IgG. Other laboratory investigations were as follows: Creatinine 0.75 mg/dL, LDH 135 U/L, BUN 18 mg/dL; (reference ranges: 0.6–1.1 mg/dL, 140‐280 U/L, 7–20 mg/dL, respectively).

The patient was seen in consultation, and a recommendation was made to treat her with steroids after discussing her case with the pathologist.

## Discussion

Formerly known as IgG4‐related systemic disease, IgG4‐related disease (IgG4‐RD) is a relatively newly discovered fibro‐inflammatory condition and is named so due to the presence of antibody subtype IgG4‐producing plasma cells, that are present in large amounts on tissue samples from involved organs. It has a characteristic histologic appearance and elevated IgG4 concentrations in the acute phase of approximately 60–70% of its patients 1, 2. The condition can be identified by tumefactive lesions, a dense lymphoplasmacytic infiltrate rich in IgG4‐positive plasma cells, and storiform fibrosis.

IgG4‐RD can virtually occur in any organ of the body with the most common sites as the salivary glands, periorbital tissues, pancreas, biliary tree, kidneys, lungs, lymph nodes, breast, aorta, thyroid, prostate, and skin 3, 4. From our review of literature, this is the second known occurrence of IgG4‐RD in the musculature 5. In light of these rare incidences, patients with associated autoimmune diseases, presenting with myalgias of unknown origin that may be accompanied by swelling, discomfort, or general unease in a particular group of muscle should prompt the physician to include IgG4‐RD in the evaluation of their differentials. The histopathological characteristics bear remarkable similarity across organs regardless of the site of disease, therefore becoming analogous to diseases like sarcoidosis. Several previously known diseases are now considered to be manifestations of IgG4‐RD: Type 1 autoimmune pancreatitis, mediastinal fibrosis, Küttner's tumor (affects the submandibular glands), Mikulicz's disease (involvement of the lacrimal and salivary glands), Riedel's thyroiditis, Ormond's disease (retroperitoneal fibrosis), and inflammatory pseudotumor are now regarded as forms of IgG4‐RD 4.

IgG4‐RD has been labeled as a slow condition as usually symptoms tend to be mild in spite of considerable organ destruction, if any. People are often described feeling relatively well at the time of diagnosis, although some may give a history of weight loss. Pain is usually not a feature of the inflammation but may occur as a secondary effect due to some form of obstruction or compression. A diagnosis is often formulated due to the presence of painless swellings or mass lesions that may lead to complications. Incidental lesions may also be detected on radiological studies and can be easily misdiagnosed as a malignancy.

Several genetic studies suggest the involvement of several human leukocyte antigen (HLA) and non‐HLA haplotypes/genotypes that may be associated with susceptibility to IgG4‐RD or to disease relapse post therapy 6. Among the cohort of autoantibodies found to date, autoantibodies against carbonic anhydrase II and lactoferrin are most commonly noted in the serum of IgG4‐RD patients 7, 8. Studies have shown molecular mimicry between *Helicobacter pylori* and constituents of pancreatic epithelial cells suggesting gastric *H. pylori* infection may set off autoimmune pancreatitis in genetically predisposed individuals via cross‐reactivity of the antibodies, yet autoimmunity has not been completely proved. Antibodies directed against potential auto‐antigens to some degree, may be associated with systemic manifestations of IgG4‐RD 6. It has also been postulated that T2‐helper cells are a predominant cell type in IgG4‐RD, while transforming growth factor‐b and interleukin‐10 have roles in IgG4 class switch and fibroplasia 6.

Radiographic features are generally nonspecific and may not provide a reliable distinction between IgG4‐RD and malignancy. A majority of patients with IgG4‐RD have elevated serum IgG4 levels of varying ranges with approximately 30% of patients having normal serum IgG4 despite classic histopathological and immunohistochemical features 4, 9. Data with regard to the use of serial IgG4 measurement as an indicator of disease activity is mixed 10. Despite the reduction of IgG4 concentrations with glucocorticoid treatment in a majority of patients, they still tend to remain above normal values 9. Kamisawa et al. 11 demonstrated that IgG4 levels failed to normalize in 63% (115 of 182 patients) treated with glucocorticoids. It also has been shown that the disease can remain in remission in some patients despite persistent elevations of serum IgG4 concentrations, with only 30% of patients having persistent elevation of serum IgG4 concentrations suffering relapses 11.

Induction and maintenance of remission are the main treatment goals to prevent progression of fibrosis and organ damage. All cases of symptomatic IgG4‐RD warrant treatment though some cases of asymptomatic IgG4‐RD may require early management to prevent severe complications like aoritis, pericarditis, and pachymeningitis 12.

Glucocorticoids are the recommended first‐line agents for induction unless contraindications exist resulting in rapid improvements of symptoms and a resolution of radiographic findings. Steroid‐induced flares are not uncommon. Although their efficacy has never been tested in clinical trials, rituximab, mycophenolate mofetil, methotrexate, and azathioprine are used frequently as glucocorticoid‐sparing agents or remission‐maintenance drugs after glucocorticoid‐induced remissions 4, 12. Other therapeutic possibilities, such as bortezomib, that are known to target plasma cells, warrant further exploration as well.

Bortezomib, an excellent proteasome inhibitor has been a novel drug which is currently approved for and used in the management of relapsed leukemias and multiple myelomas in the posttransplant setting. Several preclinical trials showed antimyeloma effects of bortezomib including the disruption of the cell cycle and apoptosis induction, alteration of the bone marrow microenvironment, and inhibition of nuclear factor kappa B (NF*κ*B) 13. Due to its unique mechanism of action, bortezomib has been shown to induce responses in patients with poor cytogenetics, and in previously refractory patients. Although clinical trials have yet to be established for the use of this drug in the management of IgG4‐RD, Khan et al. 14 demonstrated the successful use of bortezomib in a patient with Hyper IgG4.

## Conclusion

IgG4‐RD encompasses a large number of medical disorders previously referred to as confined to a single organ. As a recently recognized condition, links among the increased quantities of IgG4 plasma cells, varying quantities of serum IgG4, and its histological features needs to be determined. Despite being a rare case in itself, to the knowledge of the authors, this is the second known case of IgG4‐RD to occur in the skeletal muscles.

## Conflict of Interest

None declared.
